# A Comparison Study of the Caecum Microbial Profiles, Productivity and Production Quality of Broiler Chickens Fed Supplements Based on Medium Chain Fatty and Organic Acids

**DOI:** 10.3390/ani11030610

**Published:** 2021-02-26

**Authors:** Agila Dauksiene, Modestas Ruzauskas, Romas Gruzauskas, Paulina Zavistanaviciute, Vytaute Starkute, Vita Lele, Dovile Klupsaite, Jolita Klementaviciute, Elena Bartkiene

**Affiliations:** 1 Institute of Animal Rearing Technologies, Lithuanian University of Health Sciences, Tilzes str. 18, LT-47181 Kaunas, Lithuania; paulina.zavistanaviciute@lsmuni.lt (P.Z.); vytaute.starkute@lsmuni.lt (V.S.); vita.lele@lsmuni.lt (V.L.); dovile.klupsaite@lsmuni.lt (D.K.); jolita.klementaviciute@lsmuni.lt (J.K.); elena.bartkiene@lsmuni.lt (E.B.); 2 Department of Anatomy and Physiology, Lithuanian University of Health Sciences, Tilzes str. 18, LT-47181 Kaunas, Lithuania; modestas.ruzauskas@lsmuni.lt; 3 Microbiology and Virology Institute, Lithuanian University of Health Sciences, Tilzes str. 18, LT-47181 Kaunas, Lithuania; 4 Department of Food Sciences and Technology, Kaunas University of Technology, Radvilenu str. 19, LT-50254 Kaunas, Lithuania; romas.gruzauskas@ktu.lt; 5 Department of Food Safety and Quality, Lithuanian University of Health Sciences, Tilzes str. 18, LT-47181 Kaunas, Lithuania

**Keywords:** poultry, microbiome, caecum microbial profiles, productivity characteristics, production quality

## Abstract

**Simple Summary:**

The ban of growth promoters in poultry farming in the European Union has resulted in the development of alternatives. Among these alternatives, medium chain fatty acids (MCFAs) or organic acids (OAs) are considered to be suitable for in-feed use. However, their effect on microbiota modulation and the meat quality of broiler chickens are still under-investigated. The aim of this study was to estimate the influence of MCFAs and OAs supplements on the caecum microbial profiles, productivity and production quality characteristics of broiler chickens. The 42-days experiment was conducted using 900-day-old broiler chickens, allocated into three groups, consisting of 300 birds per group. The results indicated that the addition of OAs results in a more appropriate environment in the caecum for beneficial microorganisms rather than diets supplemented with MCFAs. These positive changes led to a higher efficiency of poultry productivity (higher body weight and lower mortality); however, for most of the analysed broilers’, technological parameters were not considerably influenced by treatments.

**Abstract:**

The aim of this study was to evaluate the influence of medium chain fatty acids (MCFAs) and organic acids (OAs) supplements on the caecum microbial profiles, productivity and production quality characteristics of broiler chickens (BCs). BC (900 chicks) were attributed to three groups: (i) control; (ii) MCFAs group (BCs fed with feed supplemented with MCFAs); (iii) OAs group (BCs fed with feed supplemented with OAs). Broilers were slaughtered at the end of the trial (42 days old), and the caecum microbial profiles, productivity and production quality characteristics were analysed. Supplementation with OAs resulted in a more appropriate environment in the caecum for beneficial microorganisms than with a diet supplemented with MCFAs. This was supported by data on the presence of higher amounts and an increased species variety of probiotic bacteria (*Lactobacillus* and *Bifidobacterium*) in the caecum of birds. The above-mentioned changes of the caecum microbiota led to significantly higher villus height (*p* = 0.003) of the OAs broiler group and significantly lower crypt depth (*p* = 0.037). Notwithstanding the significant increase of acetic, propionic, isobutyric, butyric, isovaleric, and valeric acids that were established in caecum samples from the MCFAs group, better parameters of broiler production performance (higher body weight and lower mortality) and carcass traits (higher both thigh and shin muscles with skin and bone weight; both shin muscles without skin and bone weight; abdominal fat yield) were found in the OAs-treated group. For chemical, physical and technological characteristics of breast meat samples, increased yellowness and water holding capacity by 14.7% and 2.3%, respectively, were found in MCFAs group samples. A more appropriate environment in the caecum for beneficial microorganisms could be obtained when BCs were fed with OAs supplement, comparing to MCFAs, and these positive changes were associated with higher efficiency of poultry production.

## 1. Introduction

The effectiveness of poultry production is influenced by several factors, the most important of which are genetic, environment, nutrition and management. In terms of nutrition, it should be balanced for a better growth efficiency of broilers, as well as higher production quality and safety. In the past decades, antibiotic growth promoters were applied to improve feed utilisation and health conditions in poultry species [1]. However, the use of antibiotic growth promoters in animal production led to an increase in the amount of antimicrobial-resistant bacteria and infections that are difficult to treat. In 2006, the European Union imposed a complete ban on the use of growth promoters in poultry feeds. As a consequence, the development of alternatives to growth promoters received considerable attention [2]. Among these alternatives, organic acids (OAs) are considered to be suitable for in-feed use. These compounds, usually short chain fatty acids (SCFAs), selectively stimulate the favourable growth or activity of beneficial bacterial species and the death of harmful bacteria inhabiting the digestive tract of poultry [2]. Butyric acid is one of the acids that has been successfully used in poultry production. It is involved in the development of gut wall tissues and modulates the growth of symbiotic intestinal microbiota as well as improving immunity in broilers [3,4]. Butyrates are readily transformed into butyric acid within the digestive tract and are considered safe for animals and humans [5]. Butyrate beneficially reduces the concentrations of total circulating triglycerides and cholesterol in broilers [6]. Other organic acids that are used in poultry production are acetic, propionic, lactic, malic, tartaric fumaric, formic, sorbic and some other acids [7]. Their effect on microbiota modulation is still under-investigated. 

Medium chain fatty acids (MCFAs) are among the most promising as an alternative to antimicrobial usage in poultry [8]. Caproic, caprylic, or capric MCFAs are digested and absorbed faster than long-chain fatty acids (LCFAs) and may be very useful when the digestion, absorption, or transport of dietary fat is defective [9]. MCFAs have been shown to be good alternatives for nutritional antibiotics in piglets due to their high antibacterial activity, and they enter cells un-dissociated [10]. MCFAs inhibit the production of lipases by bacteria [10]. Furthermore, the antibacterial potency of MCFAs is believed to exceed that SCFAs [11]. During the first week, MCFAs are important players in the build-up and maintenance of the poultry’s health [11].

The supplementation of SCFAs and MCFAs in the broiler diet is beneficial, acting to lower serum cholesterol, abdominal fat, and thigh meat fat percentage; this might be attributed to the ability of SCFAs and MCFAs to improve meat quality [9]. It was previously reported that OAs could improve poultry growth performance [12,13], but other studies have not reported the same significant results [14,15]. The main explanation for these differences is the heterogenicity of conditions in which each experiment was carried out, differing in the chemical structure of the utilised acid and in the supplementation form (mixed or not), as well as in the sanitary challenge conditions, buffering capacity of feeds, and feeds’ dietary nutritional value, among other factors [16].

This study investigated the hypothesis that dietary OAs or MCFAs may support intestinal health by affecting the intestinal microbiota, intestinal antimicrobial activity, following enhanced digestibility of nutrients, thus improving growth performance, giving lower feed conversion ratio and mortality, better carcass traits and meat quality of broiler chickens. Thus, the aim of the present study was to investigate the influence of MCFAs and OAs supplements on the caecum microbial profiles, productivity and production quality characteristics of broiler chickens.

## 2. Materials and Methods

### 2.1. Animals and Housing

All animal procedures were conducted according to the EU Directive 2010/63/EU of the European Parliament the Council of 22 September 2010 on the protection of animals used for scientific purposes and Requirements for the Keeping, Maintenance and Use of Animals Intended for Science and Education Purposes, was approved by order of the Lithuanian Director of the State Food and Veterinary Service, 31/10/2012, No. B1-866. The study was conducted at a poultry farm in Kaisiadorys district (Kaisiadorys, Lithuania) and the Institute of Animal Rearing Technologies Lithuanian University of Health Sciences (Kaunas, Lithuania). An experiment with a duration of 42 days was conducted using a total of 900 day-old Ross 308 broiler chickens. The broiler chickens were kept on deep litter. The density of broilers was 16 units per 1 m^2^. Drinking water and compound feed were available *ad libitum* throughout the trial. The electric light illuminated the aviary over 24 h. The aviary temperature was between 32 °C (at the beginning of the trial) and 20 °C (at the end of trial) and followed the Ross 308 cross recommendations for broiler chickens. In the poultry house the relative humidity was 60–70%. The birds were vaccinated against infectious bursal disease (Gumboro disease), Newcastle disease, and avian infectious bronchitis. Antibiotic treatment was not applied to the birds.

### 2.2. Experimental Design and Diets 

The broiler chickens (BCs) were reared to 42 days of age. The BCs were randomly distributed into three groups, with each group consisting of 300 birds with six replicates per group. Three dietary treatments were compared: (i) basal diet without supplementation, (ii) basal diet containing MCFAs supplement (MCFAs group), and (iii) basal diet containing organic acids composition supplement (OAs group). A phase feeding (pre-starter, starter, grower, and finisher) was applied. MCFAs (C_6_-C_8_-C_10_-C_12_) at the level of 0.2% of feed were included in the diet of the MCFAs group. The feed of the OAs group was supplemented with OAs at the level of 0.2% feed. OAs composition used in the experiment consisted of formic acid (38%), lactic acid (16%), propionic acid (11%), butyric monoglyceride, propionic and benzoic acids (11%), ammonium formate (9%), ammonium propionate (6%), acetic acid (6%), citric acid (1.5%), and sorbic acid (1.5%). The feed of all periods consisted of a corn-soybean meal-based diet and was formulated according to the nutritional requirements prescribed in the Ross 308 management guide (*Avigen^®^*, revised in 2019; http://eu.aviagen.com/tech-center/download/1339/Ross308-308FF-BroilerPO2019-EN.pdf, accessed on 26 January 2021). Table 1 lists the calculated values of the compound feed for control, the MCFAs and OAs chicken groups. The crude protein was analysed by using official method 2001.11, crude fat by official method 945.16, crude fibre by official method Ba 6a-05, ash by official method 942.05, calcium by official method 968.08D, and total phosphorus by official method 965.17 (https://www.aoac.org/official-methods-of-analysis-21st-edition-2019, accessed on 26 January 2021) [17].

### 2.3. Metagenomics and Microbial Profiling Analysis

Before the experiment, faeces (meconium) from one-day-old chicks representing control, OAs and MCFAs groups were collected from ten birds in each group. The amount of 0.1 g of faeces was placed onto a sterile Petri dish from each bird separately. Then the content from each chicken group was placed in a sterile tube and mixed using a single-tube mixer, making a single pooled sample for each group. A similar procedure, using 10 chickens in each group, was used at the end of the experiment during slaughtering (42 day-old chicken), making representative pooled samples from caeca content. Pooled samples were kept in DNA/RNA Shield 1:10(R1100-250, Zymo Research, Irvine, CA, USA) at −70 °C before DNA extraction, which was made using the faecal DNA MiniPrep kit (D6010, Zymo Research, Irvine, CA, USA) and thereafter purified and concentrated using a DNA Clean and Concentrator-25 kit (Zymo Research, Irvine, CA, USA) to produce at least 50 ng/µL DNA. Initial control of the DNA was performed using NanoDrop 2000 spectrophotometer (Thermo Scientific, Waltham, MA, USA), whereas further quality control was performed at the independent enterprise BaseClear (Leiden, The Netherlands) using Qubit 4 fluorometer (Thermo Scientific, Waltham, MA, USA). Metagenomic libraries were prepared, sequenced, quality controlled, and assembled in the independent service laboratory (Baseclear, Netherlands). Short-paired sequence reads were generated using the Illumina MiSeq system (Illumina, San Diego, CA, USA) and converted into FASTQ files using the BCL2FASTQ pipeline version 1.8.3. Quality trimming was applied based on Phred quality scores. Subsequently, the Illumina paired reads were merged into single reads (so-called “pseudoreads”) through sequence overlap. Chimeric pseudoreads were removed, and the remaining reads were aligned to a combination of the GreenGenes and RDP 16S gene databases. Based on the alignment scores of the pseudoreads, the taxonomic classes were assigned by associating each pseudoread to the best matching Operational Taxonomic Unit (OTU). ZymoBIOMICS Microbial Community Standard (D6300, Zymo Research, Irvine, CA, USA) was used for microbiome profiling quality control. The results of taxonomic classification were presented on the interactive online platform of the independent enterprise BaseClear. The variety of *Lactobacillus* species were analysed, taking the data from sequencing analysis data. *Lactobacillus* species variety and the prevalence of each species were compared among all the chicken groups.

### 2.4. Histomorphology of the Duodenum 

At the end of the trial, 10 broilers with average weight from each group were taken from the slaughterhouse to evaluate the histomorphology of the duodenum. The entire gastrointestinal tract was removed, and the duodenum separated. A histomorphological analysis of the duodenum was performed [18]. Segments measuring 2 cm in length were cut from the mid-points of the duodenum, flushed with cold saline, fixed in 10% buffered formalin, and stained with haematoxylin and eosin. Histological sections were examined by microscope with the deconvolution imaging analysis system (VayTek^®^, Fairfield, IA, USA). Villus height (VH) (from the tip of the villus to the top of the lamina propria) and crypt depth (CD), from the base to the region of transition between the crypt and villus in the duodenum, were determined. Measurements of 10 complete villi for VH and associated crypts for CD were taken from the duodenum, and the average of these values was used for statistical analysis. The ratio between VH and CD was described as VH/CD.

### 2.5. Short-Chain Fatty Acids (SCFAs) Evaluation in Broilers Caecum Chymus 

At the end of the trial, 10 broilers from each group were taken from the slaughterhouse to determine caecal SCFAs (acetic, propionic, butyric, isovaleric, valeric, isocaproic, caproic, and n-heptanoic acids) concentrations. The caecal chymus samples were frozen immediately after collection and stored at −20 °C until analysis. One gram of sample was thawed and suspended in at least 5 mL of water and homogenised for about 3 min, resulting in a 17% (*w*/*w*) faecal suspension. After that, the pH of the suspension was adjusted to 2–3 by adding 5 m HCl and then kept at room temperature for 10 min during occasional shaking. The suspension was transferred into a polypropylene tube and centrifuged for 20 min at 5000 rpm, giving a clear supernatant. The internal standard, 2-ethylbutyric acid solution, was spiked into the supernatant at a final concentration of 1 mm, and the supernatant was injected in the Gas Chromatograph GC-2010 Plus (Shimadzu corp., Kyoto, Japan) with a Mass Spectrometer GCMS-QP2010 (Shimadzu corp., Kyoto, Japan) for analysis [19].

### 2.6. Measurements of the Productivity Parameters 

The BCs were monitored daily. Body weight (g) and mortality (%) were recorded in 7, 14, 21, 28, 35, and 42 day-old chickens, and for the entire period for each treatment, the feed conversion ratio (FCR) (g/kg) was calculated by the formula: Input of feed / wight gained by the animal.

### 2.7. Evaluation of Broiler Carcass Traits

At the end of the trial, 10 chickens with similar body weights from each group were selected for carcass trait evaluation. The carcass traits, including breast meat, thigh muscle and abdominal fat percentages, were evaluated as described by Marché (2000) [20].

### 2.8. Evaluation of Chemical, Physical and Technological Characteristics of Broiler Breast Meat Samples

The dry matter (DM) content, pH, colour coordinates, drip loss (DL), water-holding capacity (WHC), cooking loss (CL), shear force (SF, which indicates the tenderness of the meat), protein content (ISO 937:1974) [21] and intramuscular fat content of BC breast meat samples were evaluated by methods described by Mozuriene et al. (2016) [22]. 

### 2.9. Statistical Analysis

SPSS software version 15.0 (SPSS, Chicago, IL, USA) was used for statistical analysis. All analytical experiments were carried out in triplicate. The analysis of variance was used to determine whether significant differences existed between means. Differences were classified by Duncan multiple comparison test. Differences between the most prevalent bacterial species among two groups of chicken fed with different supplements were assessed using the Z-Test Calculator for two Population Proportions (Social Science Statistics, socscistatistics.com). Results were considered statistically significant at *p* ≤ 0.05.

## 3. Results

### 3.1. Microbial Profiling Analysis

**Figure 1:**
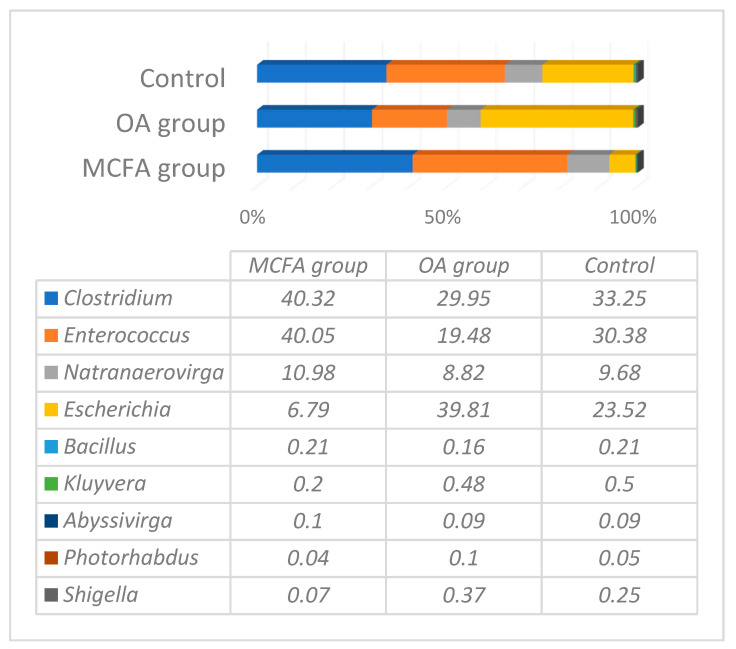
The representative microbiota at a genus level in ceca content of control, medium chain fatty acids (MCFAs) and organic acids (OAs) groups before the experiment (one-day-old chicks).

Representative microbiota at a genus level before the experiment in one-day-old chicks are presented in Figure 1. The total number of reads at the genus level was 31,986, 31,946, 29,267, and in the control, MCFAs and OAs samples groups, respectively (*p* ≤ 0.05). Nine different genera with a prevalence at least 0.1% were detected in all of the tested groups; however, only four genera were predominant, with the total prevalence being more than 98% from all OTUs. These bacteria depended to the genera *Clostridium*, *Escherichia*, *Enterococcus*, and *Natranaerovirga*. The microbial diversity among the groups was very similar, but the amount of *Enterococcus* and *Escherichia* varied significantly between the groups: the amount of *Enterococcus* was the lowest in the OAs group (19.48%), and the amount of *Escherichia* was the lowest in the MCFAs group (6.79%).

**Figure 2:**
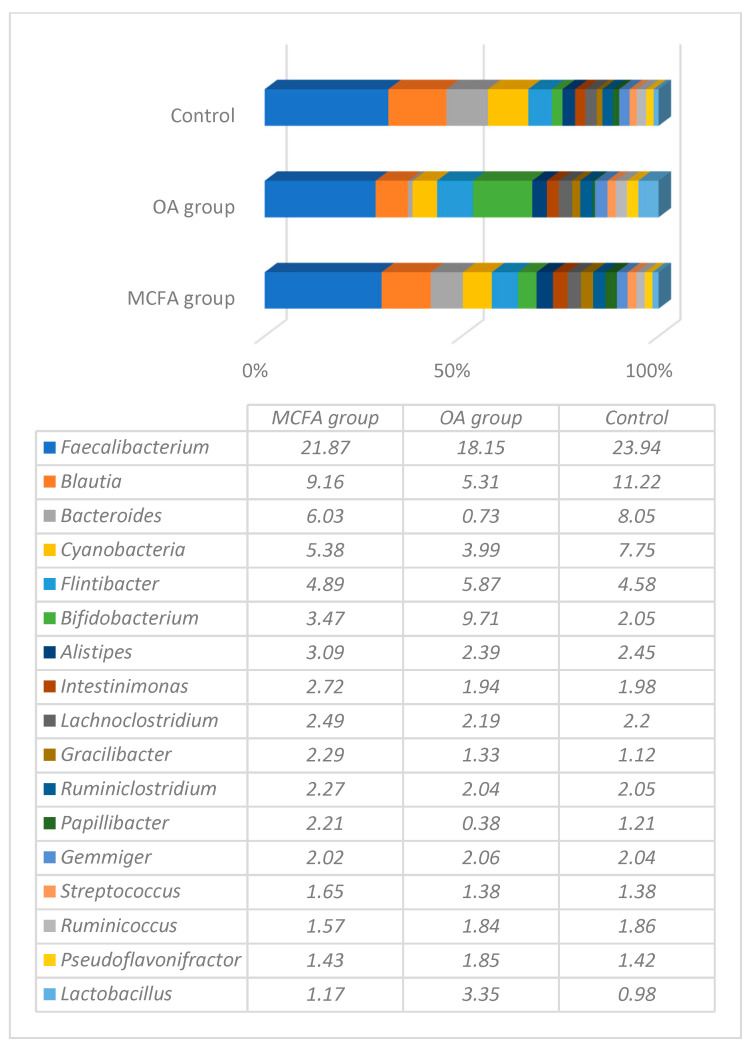
The representative microbiota at a genus level in ceca content of control and experimental groups at the end of the experiment (42-day chickens).

Microbial profiling regarding the variety of bacterial genera at the end of the experiment is presented in Figure 2. The main differences between the groups were in the number of probiotic bacteria—*Bifidobacterium* and *Lactobacillus* (with significantly reliable results (*p* ≤ 0.05) between OAs and two other groups. The amount of *Bifidobacterium* was 9.71%, 3.49%, and 2.05%, whereas the amount of *Lactobacillus* was 3.35%, 1.17%, and 0.98% in the OAs, MCFAs and control groups, respectively. The OAs group harboured fewer bacteria of the *Blautia*, *Pappilibacter* and particularly *Bacteroides* genera (0.73% vs 6.03% and 8.05%) in comparison to the rest of the groups (*p* ≤ 0.05).

**Figure 3:**
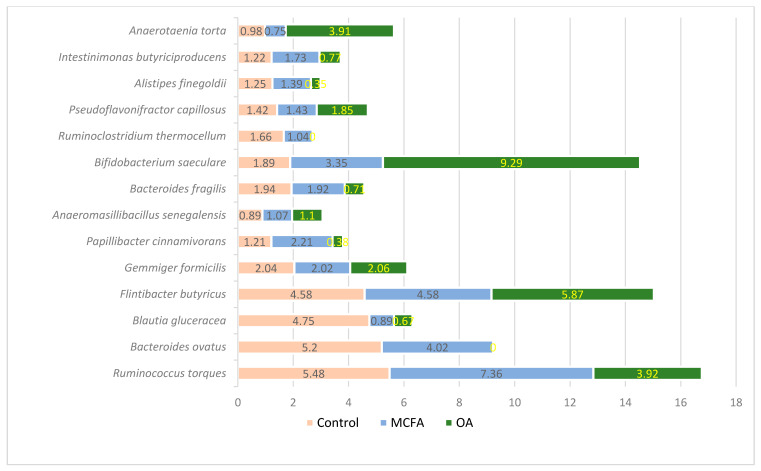
The most prevalent bacterial species (% from all species) in different chicken groups at the end of the experiment.

The composition of the most prevalent species (except *Faecalibacterium prauznitzii*) in chicken guts after the experiment is presented in Figure 3. 

The most prevalent species in all groups was *Faecalibacterium prausnitzii*, with a prevalence of 23.4%, 21.87%, and 18.15% (*p* ≤ 0.05) in the control, MCFAs, and OAs groups, respectively. The microbial profile in the MCFAs group was more similar to that of the control group. The highest differences in the OAs group in comparison with other groups was towards the number of *Anaerotaenia torta* and *Bifidobacterium saeculare*, of which the prevalence in the OAs group was significantly higher (*p* ≤ 0.05) as well in the amount of *Alistipes finegoldi*, *Rhuminoclostridium thermocellum*, and *Papillibacter cinnamivorans*, of which the prevalence in this group was significantly lower. *Bacteroides ovatus* was not detected in the OAs group. As it was detected that *Lactobaciollus* genus prevalence was higher in the OAs group, but the prevalence of a single species was less than 1% in all groups, the species variety among this genus was analysed (Table 2).

The results demonstrated a huge variety of *Lactobacillus* species in chickens’ gut. In total, 35 different species of this genus were detected, with the highest prevalence being of two species—*L. crispatus* and *L. salivarius*. The number of species found in chickens depended on the group: the largest variety was detected in the OAs group (32 species), whereas in the MCFAs and control groups, the variety was much lower (22 and 19 species, respectively).

The difference in *Bifidobacterium* species variety was also significant between the groups: twenty-one species versus seven were detected in the OAs and MCFAs groups, respectively (data not shown). The most prevalent species in both groups was *Bifidobacterium saculare*, with a prevalence of 97% and 96% in the MCFAs and OAs groups, respectively.

### 3.2. Histomorphology Parameters of Broilers Duodenum 

The duodenum villus height (VH) and crypt depth (CD) of broilers are shown in Table 3. Significantly higher villus (*p* = 0.003) and lower CD (*p* = 0.037) were established at the end of the experiment in the OAs supplement-fed group (on average, by 14.5% higher and by 13.8% lower, respectively).

### 3.3. Short-Chain Fatty Acids Concentrations in Broilers’ Caecum Chymus

Most of the analysed SCFAs concentration in broilers’ caecum chymus varied according to the supplement used for chicken feed (Table 4). Significantly higher (*p* ≤ 0.05) concentrations of acetic, propionic, isobutyric, butyric, isovaleric, and valeric acids were found in samples from the MCFAs group (by 9.8%, 2.9%, 12.1%, 2.5%, 0.3%, and 2.6%, respectively), compared with the control group. However, significant differences between the isocaproic, caproic, and n-heptanoic acids between the MCFAs or OAs groups and control group samples were not established.

### 3.4. Parameters of the Broiler Chickens’ Production Performance

The results of production performance: body weight, mortality after 7, 14, 21, 28, 35, 42 days of feeding, as well as average daily weight gain (ADWG)and feed conversion ratio (FCR) are given in Table 5. The results showed a significant increase in average body weight (*p* ≤ 0.05) in the pre-starter (first–seventh day) and overall (first–forty-second day) periods (on average, by 3.5% and 5.8%, respectively) for the OAs group compared with the control group. 

Mortality in the OAs group was significantly lower in the pre-starter (first–seventh day), starter (eighth–fourteenth and fifteenth–twenty-first days), finisher (twenty-ninth–forty-second day) and overall (first–forty-second day) periods compared with the control group. The addition of OAs had a significant influence (*p* ≤ 0.05) on FCR (first–forty-second day)—it decreased by 2.4%. 

### 3.5. Carcass Traits

Carcass traits of the BC fed with diets supplemented with MCFAs and OAs are shown in Table 6. Carcass weight, both wings and both leg muscles, with and without skin and bone weight, both thigh muscles without skin and bone weight, breast muscles without skin weight, abdominal fat, and chest ridge length were not considerably influenced by the treatments (*p* ≥ 0.05). In this study, significantly higher weights of both thigh and shin muscles with skin and bone in the OAs group, compared with the control group, were found (on average, by 8.3% and 3.0%, respectively). Also, the weight of both shin muscles without skin and bone in the OAs group was significantly higher (by 1.4% on average) compared with the control group.

Significantly higher lengths of tibia bones and the weight of carcass bones without wings and legs were established in the OAs group chickens (on average, by 5.9% and 10.3%, respectively), compared with the control group. However, significant differences between the control and OAs groups in the broilers’ breast and leg muscle yield were not found. A significantly higher abdominal fat yield was found in OAs group samples compared with the control group (by 13.8% higher).

### 3.6. Chemical, Physical, and Technological Characteristics of Broiler Breast Meat Samples

Quality parameters of the breast meat samples from different groups of broilers fed with MCFAs or OAs are given in Table 7. From all of the analysed parameters, significant differences were established between yellowness (b*) and water holding capacity (WHC) of the meat samples (*p* = 0.027 and *p* = 0.009), with b* coordinates and WHC that were by 14.7% and 2.3% higher, respectively, in the MCFAs group breast samples.

## 4. Discussion

Although the microbial analysis of the caeca of broilers was performed on hatching day, we detected more than thirty thousand reads associated with bacterial DNA. According to microbial profiling, it appears that microorganisms that started to colonise the gut of domestic birds are associated with conventional bacteria that are widespread in a close environment. The most prevalent bacterial genera within the embryo of chickens were *Lactobacillus*, *Fusobacterium*, *Megamonas*, *Bacteroides*, *Staphylococcus*, *Enterococcus*, and particularly *Pseudomonas*. Although chicks from the OAs and MCFAs groups were hatched in different flocks (but in the same poultry farm), their microbial composition at the beginning of the experiment was very similar. Up to 98% of all microbiota in both groups consisted of four genera, of which at least three were cultivable and widely widespread (*Clostridium*, *Escherichia*, and *Enterococcus*); however, the less investigated genus *Natranaerovirga* from the order Clostridiales also represented the microbial community in one-day-old chicks with a prevalence of 9–11% of the total bacterial load. Other studies have demonstrated different results in bacterial variety in one-day-old chicks; however, most of them noticed the prevalence of Enterobacteriaceae, *Clostridium*, and *Enterococcus* [23,24,25] during the first days of life in chickens. The amount of *Enterococcus* in the OAs group before the experiment was significantly lower, whereas the number of *Escherichia coli* was higher in comparing with the other groups of chicken; however, at the end of the experiment, the number of enterococci and *E. coli* in all groups were detected in only ≤10 reads (0.02–0.03%). We suggest that such differences among the groups at the beginning of the experiment had no influence on the results of microbial changes caused by using the supplemented feed. The data also are in coincidence with the findings of other authors, which prove that *E. coli* and enterococci are highly prevalent only at a very young age of chickens. 

The high diversity of microorganisms in the caeca was observed at the end of the experiment, with the highest prevalence being seen for *Faecalibacterium*. This bacterium is closely related to members of *Clostridium* cluster IV [26]. *Faecalibacterium* is among the major genera in both human and pig guts. Similarly, in the chicken gut, these genera are also among the major genera of relatively high abundance, indicating the importance of these gut microbes in both birds and mammals [27]. The study performed by Donaldson et al. (2017) [28] demonstrated that *Faecalibacterium* increased in abundance slowly and steadily over time, while others like *Enterobacter* were only abundant in the first days of life. In the study performed by Yan et al. (2017) [29], the prevalence of *Faecalibacterium* was lower in the better feed efficiency group of broilers than in the poor feed efficiency group. In our study, the prevalence of this genus was slightly lower in the OAs group compared to the MCFAs group. Recently, it was revealed that *Faecalibacterium* (and some *Clostridium)* increased their relative abundance in the chicken gut when *C*. *jejuni* colonised the chicken caecum [30]. In our study, *Campylobacter* was only detected in low amounts and probably had no influence on high amounts of *Faecalibacterium* in the caeca of birds.

The highest differences in the caeca of 42-day-old birds between the MCFAs and OAs group were among the prevalence of *Bacteroides*, *Papillibacter*, and *Blautia,* which was higher in the MCFA group, whereas *Bifidobacterium* and *Lactobacillus* were significantly more abundant in the OA group. 

*Bacteroides* and *Blautia* are involved in producing SCFAs [31]. Additionally, *Bacteroides* also play an important role in breaking down complex molecules into simpler compounds which are essential to the growth of host and gut microbiota [32,33]. *Bacteroides* and *Faecalibacterium* are suggested to be involved in the decrease of regulatory T-cell expansion and the stimulation of anti-inflammatory cytokine production [33]. However, the beneficial functions of *Bacteroides* are strain-dependent [33]. To some extent, *Bacteroides* are considered to be opportunistic pathogens, as some of them are carriers of virulence factors, such as the enterotoxigenic *B. fragilis* producing fragilysin [34]. *Papillibacter* has been reported to be isolated from the chicken gut [35,36]; however, there is still no information regarding their functions in the digestive tract of vertebrate animals. 

The abundance of *Lactobacillus*, calculated from the total amount of bacteria, was 2.9 times, and *Bifidobacterium* 2.8 times higher in the caeca of the OA group, compared with the MCFA group. This fact suggests an opinion that supplementation of the feed by organic acids supplements can alter microbial profiles in chickens with increasing populations of probiotic bacteria. It is known that in the chicken, along with the ability to improve production parameters and to limit food-borne pathogenic bacteria [37,38,39], *Lactobacillus* species have been shown to stimulate multiple aspects of the immune response [40]. The total number of different species of lactobacilli was significantly higher in the OAs group, but the most abundant species in both groups were *L. crispatus* and *L. salivarius,* which prevalence consisted of more than 50 percent of the all amount of lactobacilli detected. *L. crispastus* was originally isolated from a pouch in a chicken oesophagus and is considered to be one of the strongest H_2_O_2_ producing lactobacilli [41]. It has been proved to be the most stable and protective species compared with other lactobacilli [42]. *L. salivarius* is also considered a probiotic bacteria species that have been found to live in the gastrointestinal tract and exert a range of therapeutic properties, including the suppression of pathogenic bacteria [43]. The above-mentioned lactobacilli are successfully used as probiotics for humans, poultry, pigs, dogs, and equines and are naturally found in those species of animals and humans [44,45,46]. Supplementation of the mixture of three *L. salivarius* strains to broilers for 42 days improved body weight, body weight gain, and feed conversion ratio, reduced total cholesterol, LDL-cholesterol and triglycerides, increased populations of beneficial bacteria such as lactobacilli and bifidobacteria, decreased harmful bacteria such as *E*. *coli* and total aerobes, reduced harmful caecal bacterial enzymes such as β-glucosidase and β-glucuronidase, and improved the intestinal histomorphology of broilers [47]. It is important that 35 species of lactobacilli were detected in the gut of healthy chickens within this study, supporting the opinion that this genus of bacteria is one of the most important as a protective shield against pathogenic bacteria that we have not detected. In addition, *Campylobacter* species, which are considered to be normal or commensal microbiota in birds, and the prevalence of which in chickens usually is high (an EU baseline survey including data from 26 European Union Member States and two countries not belonging to the European Union showed that 75% of broiler batches carried *Campylobacter* in their cecal contents) [48], were almost absent. 

*Bifidobacterium* produce lactic and acetic acid in large amounts, larger than lactobacilli. Similarly to lactobacilli, Bifidobacteria partake in the stabilisation of the gastrointestinal barrier, modulation of the local and systemic immune responses, the inhibition of pathogenic invasion and promotion of the bioconversion of unavailable dietary compounds into bioactive healthy molecules [49]. 

MCFA and OA had a beneficial effect on the duodenum villus height (VH) and crypt depth (CD) of broiler chickens. The microscopic structure of VH and CD are considered among the main indicators of intestinal development influencing nutrient digestion, and higher VH, as well as lower CD, are associated with the increased absorptive area of nutrients [50]. However, no standard measurements referring to the optimal CD and/or VH have been determined to date. Changes in microbiota can affect intestinal morphology through the modification of CD, which is considered one of the main characteristics of gut health, along with productivity [51]. In this study, the results showed that OA supplementation has a significant influence on both of the analysed parameters (VH and CD) and can alter the physiological intestinal morphology and subsequently the digestion efficiency, as well as productivity and production quality characteristics of the broiler chickens.

Most of the analysed SCFAs concentrations in broilers’ caecum chymus varied according to the supplement used for chicken feed. SCFAs are metabolites of bacteria in the gut, with intestinal health depending on their concentration and proportion [52]. SCFAs are an important source of energy for enterocytes [53,54], and their concentrations in the caeca of chickens can vary in accordance with the dominant microbiota [55]. Also, it should be mentioned that metabolic cross-feeding, which is the utilisation of end-products from the carbohydrate catabolism of a given microorganism by another, also strongly influences the final balance of intestinal SCFAs [56]. This occurs mainly for the formation of butyrate from acetate or lactate, is considerably lower for butyrate conversion to propionate, and is very scarce between propionate and acetate [57]. An increase of propionate is related to an enrichment of intestinal *Bacteroidetes*/*Bacteroides* [58,59,60]. It was published that modifying microbiota with antibiotics in mice led to a strong correlation between caecum levels of SCFAs and the abundance of *Bacteroides* and other members of the phylum Bacteroidetes [61]. Also, SCFAs and OAs formed in cultures of *Bacteroides* (acetate, succinate, lactate, and propionate) depend on the type of fermentable substrates, generation time and incubation period [62,63,64]. In this study, a significant increase in acetic, propionic, isobutyric, butyric, isovaleric, and valeric acids in caecum samples from the MCFAs group could be related to the above-mentioned acids produced by dominant bacteria, as the main differences among in the OAs and MCFAs groups regarding *Bifidobacterium* and *Lactobacillus* (which was more abundant in the OA group) and *Bacteroides*, *Papillibacter* and *Blautia* (which was more abundant in the MCFAs group) were established. Two *Bacteroides* species (*B. ovatus* and *B. fragilis*) were among the most prevalent species in the MCFAs group compared with the OAs group. Usually, increased concentrations of SCFAs are interpreted as a desirable change, as they increase intestinal acidity and are associated with pathogen suppression [65]. It was published that butyrate and propionate have inhibitory effects on *Salmonella*, which is an important pathogen frequently infecting poultry flocks [66]. However, in the MCFAs group, the prevalence of *Campylobacter* species was higher (0.06% from all bacterial community) compared to the OAs group. Finally, we agree with the findings that modification of the broilers’ caecum fermentation with feed supplements can reduce the colonisation of some pathogenic bacteria by the acidic intestinal environment resulting from the increased concentration of SCFAs; however, standard concentrations of SCFAs for broilers were not declared. For this reason, it can be stated that too high concentrations of SCFAs can also reduce desirable "good" bacteria. From this point of view, evaluating the productivity and production quality of the poultry becomes very important, as they are the main indicators showing nutrient metabolism in chickens.

Similar to our findings, Sultan et al. (2015) observed that body weight and water intake of BC increased, but FCR decreased significantly with the addition of OA [67]. The other authors mentioned that OAs have a beneficial effect on FCR [68] or on both BWG and FCR [69]. In contrast, Salah et al. (2018) reported that dietary supplementation with OAs decreased feed intake and BWG [70]. Smulikowska et al. (2010) reported decreased body weight gain and feed intake of broiler chickens fed diets containing OAs blends [71]. The increase in body weight can be attributed to the increased utilisation of nutrients when OAs were applied to the feed [72]. Adil et al. (2010) reported that OAs reduces intestinal pH and inhibits the pathogenic intestinal bacteria while decreasing the quantity of toxic bacterial end-products; this improves diet protein and energy digestibility as well as increasing the body weight of BCs [13]. As mentioned by Brzoska et al. (2013), OAs have a mortality reducing and production performance enhancing effect in broiler chickens [73]. In our study, a decreased mortality of BCs was found, which can probably be associated with the antimicrobial properties of OA, especially against the pathogenic intestinal bacteria [13]. Considerable inconsistent findings reported by different authors regarding the production performance of BCs can be related to the different inclusion levels and sources of organic acids, pKa value, cross, sex and age of BCs, the composition of compound feed, buffering capacity of diets, and experimental conditions, such as the sanitation level of the environment [74].

In this study, better parameters of carcass traits (higher both thigh and shin muscles with skin and bone weight; both shin muscles without skin and bone weight; abdominal fat yield) were found in the OAs-treated group. Thigh meat has more lipids than breast meat, and the reduction in lipid content of thigh meat can be explained by SCFAs regulating the balance between FAs synthesis, FAs oxidation, and lipolysis in the body [75]; in our study, higher SCFAs concentrations were found in caecum samples from the MCFAs group. FA oxidation is activated by SCFAs, while de novo synthesis and lipolysis are inhibited [8]. Besides the receptors Ffar2 and Ffar3, which increase leptin secretion from adipocytes, AMP-activated protein kinase (AMPK) plays an important role in this regulation, and SCFAs have been shown to increase AMPK activity in liver and muscle tissues [76]. The decreasing effects of dietary MCFAs on thigh meat lipid percentage can be attributed to the intensifying leptin secretion and its action on lipid metabolism. In vitro and in vivo experiments showed that SCFAs increase leptin expression via the Ffar2-dependent pathway [77]. Leptin, an adipokine that regulates energy expenditure and feed intake, stimulates FA oxidation by increasing the AMP/ATP ratio and AMPK activity in liver and muscle tissues [78].

Among alternative additives to antibiotic growth promoters in the diet of birds are OAs, which have strong antimicrobial properties, improving the digestibility and absorption of nutrients in the diet, weight gain and feed conversion, and reducing the production of toxic substances by bacteria and desquamation of the intestinal lining; they are used in animal feed to control the growth of fungi and bacteria, benefiting the intestinal pH and consequently the absorption of minerals, especially calcium and phosphorus, which are fundamental for the growth and development of bone tissue [79]. This may be attributed to the improved digestion mechanism and the supply of more nutrients for muscle growth. In this study, metagenomic results showed that the group fed with OAs contains more diverse desirable microorganisms, compare to the MCFAs-treated group, which can have a positive influence on nutrient degradation, as well as for absorption and muscle growth.

It was published that dietary medium-chain triglyceride decreased the abdominal fat percentage in BC [80]. Experiments in other animal species showed that MCFAs reduces body fat deposition [81]. Decreasing lipid absorption, lower calorie intake, lower biosynthesis of fatty acids (FAs), and an improvement of FAs oxidation are all provided as possible mechanisms of the reduction in body fat [82]. It was also published that MCFAs and OAs have a different metabolic fate than long chain FAs because MCFAs are rapidly absorbed in the small intestine, transported to the liver as free FAs via hepatic portal circulation, and then enter the mitochondria, independent of fatty acyl-CoA-carnitine transferase [83]. The thermal effect of MCFAs and subsequent reduction in body fat deposition could be related to the production and oxidation of ketone bodies [84].

Colour is an important meat quality characteristic as it affects consumer acceptability of meat [85]. Meat colour is influenced by sex, age, muscle pigments, meat pH, pre-slaughtering conditions and processing. Also, it is important to predict the meat WHC because this is responsible for weight loss in raw, cooked and processed meats [86]. 

The data depicting the effects of MCFAs and OAs on the broiler meat quality characteristics is scarce. It was published that a blend of MCFAs (lauric, caprylic, and capric acids) had no significant influence on the colour characteristics of broiler breast meat [87] Zeiger. However, the opposite observations were found by Begum et al. (2015), who reported that dietary supplementation of MCFAs influenced the colour of broiler meat [88]. Meat yellowness can be influenced by different supplements used in chicken diets, as they influence the microbiota of birds, which induce different biodegradation processes in the gastrointestinal tract (GIT) and releases different compounds, which can have an influence on colour formation. Also, water binding capacities of meat can have an influence on meat light reflecting; in this study, moderate positive correlations between the meat yellowness and WHC were found. Increased WHC in the MCFAs group may be related to increased breast muscle yield. In this study, no significant differences in meat pH were obtained. 

## 5. Conclusions

Supplementation with OAs, comparing with MCFAs, led to higher amounts and higher species variety of probiotic bacteria (*Lactobacillus* and *Bifidobacterium*) in the caecum of BCs. The above-mentioned changes in caecum microbiota led to significantly higher OAs broiler group VH (*p* = 0.003) and lower CD (*p* = 0.037). Also, better parameters for broiler production performance (by 5.9 % higher body weight and by 0.46% lower mortality) and carcass traits (higher thighs and shins muscles with skin and bone, both shin muscles without skin and bone weight by 8.3%, 3.0%, 1.4% respectively) were found in the OAs-treated group. Notwithstanding that, significant increases in acetic, propionic, isobutyric, butyric, isovaleric, and valeric acids in the MCFAs group, caecum samples were established. Comparison of the chemical, physical, and technological characteristics of broiler breast meat samples showed significant differences between the yellowness and WHC of the different groups’ chicken breast meat samples; results that were higher by 10.4% and 2.1% for yellowness and WHC, respectively, were found in breast samples from the MCFAs group. However, most of the analysed broilers’ meat technological parameters were not considerably influenced by treatments.

## Figures and Tables

**Table 1:** Composition and calculated values of compound feed.

Ingredients and Calculated Values	Prestarter			Starter			Grower			Finisher		
	(1–7 days)			(8–21 days)			(22–35 days)			(36–42 days)		
Dietary Treatments												
Ingredients (%)	CON	MCFAs	OAs	CON	MCFAs	OAs	CON	MCFAs	OAs	CON	MCFAs	OAs
Soybean meal	39.11	39.11	39.11	35.16	35.16	35.16	26.59	26.59	26.59	25.91	25.91	25.91
Maize	30.00	30.00	30.00	30.00	30.00	30.00	30.00	30.00	30.00	35.00	35.00	35.00
Wheat	22.47	22.27	22.27	25.35	25.15	25.15	33.11	32.91	32.91	26.57	28.57	28.57
Sunflower oil	3.58	3.58	3.58	5.01	5.01	5.01	6.16	6.16	6.16	6.33	6.33	6.33
Limestone	1.64	1.64	1.64	1.52	1.52	1.52	1.23	1.23	1.23	1.22	1.22	1.22
Monocalcium phosphate	1.16	1.16	1.16	0.98	0.98	0.98	0.55	0.55	0.55	0.57	0.57	0.57
MHA	0.46	0.46	0.46	0.44	0.44	0.44	0.52	0.52	0.52	0.49	0.49	0.49
Lysine sulphate	0.43	0.43	0.43	0.42	0.42	0.42	0.69	0.69	0.69	0.66	0.66	0.66
Wheat flour	0.30	0.30	0.30	0.30	0.30	0.30	0.30	0.30	0.30	0.30	0.30	0.30
Sodium sulphate	0.20	0.20	0.20	0.17	0.17	0.17	0.15	0.15	0.15	0.14	0.14	0.14
L-Threonine	0.19	0.19	0.19	0.19	0.19	0.19	0.21	0.21	0.21	0.19	0.19	0.19
Sodium chloride	0.18	0.18	0.18	0.18	0.18	0.18	0.20	0.20	0.20	0.20	0.20	0.20
Choline chloride	0.08	0.08	0.08	0.08	0.08	0.08	0.08	0.08	0.08	0.08	0.08	0.08
Coccidiostatic	0.05	0.05	0.05	0.05	0.05	0.05	0.06	0.06	0.06			
*Phytase* EC 5L (liquid)	0.01	0.01	0.01	0.01	0.01	0.01	0.01	0.01	0.01	0.01	0.01	0.01
*Rovabio Advance* L2	0.01	0.01	0.01	0.01	0.01	0.01	0.01	0.01	0.01	0.01	0.01	0.01
Premix	0.13	0.13	0.13	0.13	0.13	0.13	0.13	0.13	0.13	0.12	0.12	0.12
OAs			0.20			0.20			0.20			0.20
MCFAs		0.20			0.20			0.20			0.20	
**Calculated Values (%)**												
ME_N_ (MJ/kg)	12.43	12.43	12.43	12.56	12.56	12.56	13.11	13.11	13.11	13.23	13.23	13.23
Crude protein	20.02	20.00	20.00	19.02	19.00	19.00	18.02	18.00	18.00	17.52	17.50	17.50
Crude fat	6.04	5.43	5.45	7.18	6.84	6.86	8.59	8.23	8.25	8.40	8.34	8.37
Crude ash	6.64	6.56	6.62	6.04	6.07	6.13	5.22	5.21	5.28	5.13	5.13	5.16
Crude fiber	2.40	2.60	2.59	2.52	2.50	2.53	2.80	2.80	2.82	2.78	2.80	2.79
Ca	0.89	0.89	0.89	0.78	0.78	0.78	0.74	0.74	0.74	0.69	0.69	0.69
P	0.68	0.68	0.68	0.62	0.62	0.62	0.53	0.53	0.53	0.53	0.53	0.53
*Av.* P	0.46	0.46	0.46	0.44	0.44	0.44	0.41	0.41	0.41	0.38	0.38	0.38
Na	0.17	0.17	0.17	0.16	0.16	0.16	0.16	0.16	0.16	0.16	0.16	0.16
K	1.00	1.00	1.00	0.94	0.94	0.94	0.85	0.85	0.85	0.84	0.84	0.84
Cl	0.18	0.18	0.18	0.16	0.16	0.16	0.18	0.18	0.18	0.18	0.18	0.18
Lysine	1.20	1.20	1.20	1.19	1.19	1.19	1.07	1.07	1.07	1.08	1.08	1.08
*Av.* Lysine	1.16	1.16	1.16	1.08	1.08	1.08	0.96	0.96	0.96	0.93	0.93	0.93
Methionine	0.51	0.51	0.51	0.48	0.48	0.48	0.46	0.46	0.46	0.44	0.44	0.44
*Av.* methionine	0.48	0.48	0.48	0.45	0.45	0.45	0.42	0.42	0.42	0.40	0.40	0.40
Methionine + cysteine	1.03	1.03	1.03	0.95	0.95	0.95	0.90	0.90	0.90	0.85	0.85	0.85
*Av.* methionine + cysteine	0.90	0.90	0.90	0.82	0.82	0.82	0.79	0.79	0.79	0.73	0.73	0.73
Tryptophane	0.20	0.20	0.20	0.18	0.18	0.18	0.16	0.16	0.16	0.15	0.15	0.15
*Av.* Tryptophane	0.18	0.18	0.18	0.16	0.16	0.16	0.15	0.15	0.15	0.14	0.14	0.14

ME_N_—metabolic energy; MHA - Methionine hidroxi-analogue. CON—control group; MCFAs—broiler group fed with Medium Chain Fatty Acids; OAs—broiler group fed with Organic Acids. The premix provided per 1 kg of diet: 1st–21st d of age: Retinyl acetate (Vitamin A) 11.995 IU, cholecalciferol (Vitamin D3) 4.999 IU, DL-α-tocopheryl acetate (Vitamin E) 90 IU, Thiamine (Vitamin B1) 2.5 mg, riboflavin (Vitamin B2) 8.0 mg, niacin (Vitamin B3) 55.0 mg, panthotenic acid (Vitamin B5) 15.00 mg, pyridoxine (Vitamin B) 5.0 mg, folic acid 1.75 mg, vitamin B12 30 μg, Mn 112.49 mg, Zn 99.99 mg, Fe 40.00 mg, Cu 16.00 mg, I, 2.00 mg, Se 0.50 mg. 22nd–42th d of age: Retinyl acetate (Vitamin A) 11.000 IU, cholecalciferol (Vitamin D3) 5.000 IU, DL-α-tocopheryl acetate (Vitamin E) 90 IU, thiamine (Vitamin B1) 2.5 mg, riboflavin (Vitamin B2) 7.0 mg, niacin (Vitamin B3) 55.0 mg, panthotenic acid (Vitamin B5) 15.00 mg, pyridoxine (Vitamin B6) 4.0 mg, folic acid 1.75 mg, vitamin B12 25 μg, Mn 112.50 mg, Zn 99.97 mg, Fe 39.99 mg, Cu 16.0 mg, I 1.98 mg, Se 0.52 mg.

**Table 2:** *Lactobacillus* species prevalence among chicken groups at the end of the experiment.

*Lactobacillus* Species	Chicken Groups and Amount of *Lactobacillus* Species, %		
	CON	MCFAs	OAs
*L. salivarius*	34	30	19
*L. crispatus*	22	28	39
*L. kitasatonis*	12	9	8
*L. reuteri*	8	7	7
*L. johnsonii*	14	11	7
*–L. vaginalis*	0.8	0.9	6
*L. agilis*	1	0	3
*L. acidophilus*	1	0	2
*L. helveticus*	0.1	0.6	2
*L. delbrueckii*	0	2	1
*L. jenseni*	1	2	0.9
*L. avarius*	0.4	1	0.9
*L. rogosae*	0	0	0.7
*L. ingluviei*	0	0	0.4
*L. ruminis*	0	0.6	0.4
*L. mucosae*	0	0.3	0.3
*L. fermentum*	0.2	0.3	0.2
*L. acidopiscis*	0	0	0.2
*L. taiwanensis*	0	0.9	0.2
*L. gassen*	0	0	0.2
*L. gallinarum*	0	0	0.2
*L. amilolyticus*	0.4	0	0.2
*L. animalis*	0.4	0.9	0.1
*L. porcinae*	0	0	0.1
*L. iners*	0	0.3	0.1
*L. casei*	0	0	0.1
*L. oris*	2	0.9	0.1
*L. pontis*	1	0.3	0.1
*L. kafiranofaciens*	0	0	0.1
*L. frumenti*	0.2	0	0.1
*L. kisonensis*	0	0	0.1
*L. acidophilus*	0	2	0.1
*L. amylovorus*	0.4	0.3	0
*L. hellongjiangensis*	0.4	0.3	0
*L. gasseri*	0	0.3	0
unclasified	0.6	0.9	0.1
**Number of species**	19	22	32

CON—control group; MCFAs—broiler group fed with Medium Chain Fatty Acids; OAs—broiler group fed with Organic Acids.

**Table 3:** Broilers duodenum villus height (VH), crypt depth (CD), and VH:CD.

Dietary Treatments		
CON	MCFAs	OAs
Broilers duodenum villus height (VH), µm		
2467.7 ± 374.5 ^a^	2499.7 ± 380.3 ^a^	2824.6 ± 412.1 ^b^
Broilers duodenum crypt depth (CD), µm		
585.3 ± 84.7 ^a^	511.7 ± 136.2 ^a^	504.3 ± 99.5 ^b^
Broilers duodenum villus height and crypt depth ratio		
4.22	4.89	5.60

^a,b^—means with dissimilar letters varied significantly. *n* = 3 (replicates of analysis). Means of 10 birds per treatment. Birds were randomly selected and euthanised at 42 days of age. CON—Control group; MCFAs—Medium Chain Fatty Acids; OAs—Organic Acids.

**Table 4:** Short cahin fatty acids (SCFAs) concentration in broilers caecal chymus.

Short-Chain Fatty Acids Concentration in Broilers Caecum, (µmol/g)									
Groups	Acetic	Propionic	Isobutyric	Butyric	Isovaleric	Valeric	Isocaproic	Caproic	n-Heptanoic
CON	42.25 ± 0.62 ^a^	9.84 ± 0.27 ^a^	4.30 ± 0.12 ^a^	6.72 ± 0.18 ^a^	3.16 ± 0.06 ^a^	3.82 ± 0.03 ^a^	1.21 ± 0.06	0.73 ± 0.23	0.66 ± 0.01
MCFAs	46.39 ± 0.17 ^b^	10.13 ± 0.08 ^b^	4.82 ± 0.15 ^b^	6.89 ± 0.11 ^b^	3.17 ± 0.08 ^b^	3.92 ± 0.07 ^b^	1.26 ± 0.06	0.98 ± 0.29	0.66 ± 0.01
OAs	39.42 ± 1.47 ^a^	9.19 ± 0.17 ^a^	4.61 ± 0.07 ^a^	6.33 ± 0.16 ^a^	3.12 ± 0.09 ^a^	3.79 ± 0.10 ^a^	1.28 ± 0.03	0.82 ± 0.01	0.66 ± 0.01
*p*	0.01	0.001	0.01	0.001	0.01	0.01	ns	ns	ns

^a^^,b^—means with dissimilar letters varied significantly. The significance was defined at *p* ≤ 0.05. ns—not significant. *n* = 3 (replicates of analysis). Means of 10 birds per treatment. Birds were randomly selected and euthanised at 42 days of age. CON group—control group; MCFAs—broiler group fed with Medium Chain Fatty Acids; OAs—broiler group fed with Organic Acids.

**Table 5:** Broiler production performance.

Growth Performance Parameters	Dietary Treatments			
	CON	MCFAs	OAs	*p*
0 day				
Body weight, g	39.50 ± 0.35	39.50 ± 0.39	39.50 ± 0.35	ns
period (1–7 days)				
Body weight, g	161.67 ± 2.04 ^a^	165.33 ± 0.71 ^a^	167.33 ± 0.64 ^b^	0.0001
Mortality, %	0.87 ± 0.19 ^a^	0.72 ± 0.02 ^a^	0.54 ± 0.01 ^b^	0.0001
8–14 days				
Body weight, g	421.00 ± 0.71 ^a^	432.00 ± 1.22 ^b^	418.00 ± 1.58 ^a^	0.0001
Mortality, %	1.40 ± 0.12 ^a^	1.27 ± 0.07 ^a^	0.90 ± 0.12 ^b^	0.001
15–21 days				
Body weight, g	836.67 ± 4.02 ^a^	846.67 ± 4.43 ^b^	836.33 ± 1.98 ^a^	0.01
Mortality, %	2.01 ± 0.04 ^a^	1.58 ± 0.01 ^a^	1.27 ± 0.02 ^b^	0.0001
22–28 days				
Body weight, g	1443.00 ± 1.5	1459.33 ± 1.8	1458.00 ± 0.7	ns
Mortality, %	1.95 ± 0.19	1.69 ± 0.03	1.50 ± 0.16	ns
29–42 days				
Body weight, g	2043.67 ± 1.7 ^a^	2053.11 ± 2.1 ^b^	2031.0 ± 1.1 ^a^	0.001
Mortality, %	1.92 ± 0.06 ^a^	1.85 ± 0.02 ^a^	1.61 ± 0.01 ^b^	0.001
1–42 days				
Body weight, g	2420.67 ± 1.6 ^a^	2483.0 ± 2.7 ^a^	2562.3 ± 1.5 ^b^	0.0001
Mortality, %	1.63 ± 1.9 ^a^	1.53 ± 2.1 ^a^	1.17 ± 2.1 ^b^	0.0001
FCR, g/ kg	1.65 ± 0.05 ^a^	1.68 ± 0.01 ^a^	1.61 ± 0.01 ^b^	0.010

^a,b^—means with dissimilar letters varied significantly. The significance was defined at *p* ≤ 0.05. ns—not significant. FCR—Feed conversion ratio. CON group—control group; MCFAs—broiler group fed with Medium Chain Fatty Acids; OAs—broiler group fed with Organic Acids.

**Table 6:** Carcass traits of broiler chickens fed with diets supplemented with MCFAs and OAs.

Carcass Traits	CON	MCFAs	OAs	*p*
Carcass weight, g	2043.48 ± 99.0	2055.3 ± 172.3	2106.9 ± 238.9	ns
Both wings weight, g	191.44 ± 6.8	197.4 ± 8.3	202.9 ± 17.2	ns
Both legs muscle with skin and bone, g	534.98 ± 37.1	568.7 ± 56.0	586.8 ± 58.4	ns
Both thigh muscles with skin and bone, g	293.44 ± 26.2 ^a^	305.4 ± 34.8 ^a^	317.7 ± 39.2 ^b^	0.01
Both shin muscles with skin and bone, g	261.54 ± 22.1 ^a^	263.1 ± 26.8 ^a^	269.5 ± 27.8 ^b^	0.001
Both legs muscle without skin and bone, g	375.06 ± 32.7	395.0 ± 42.2	410.6 ± 50.0	ns
Both thigh muscles without skin and bone, g	204.42 ± 24.9	231.0 ± 30.7	237.5 ± 40.2	ns
Both shin muscles without skin and bone, g	170.64 ± 19.8 ^a^	163.9 ± 14.8 ^a^	173.0 ± 13.1 ^b^	0.01
Breast muscles without skin, g	573.48 ± 64.1	651.6 ± 57.1	630.8 ± 68.2	ns
Abdominal fat weight, g	29.06 ± 4.6	18.8 ± 7.2	21.6 ± 4.2	ns
Chest ridge length, cm	10.26 ± 0.4	13.1 ± 2.1	14.3 ± 1.6	ns
Length of *Femur* bone, cm	7.60 ± 0.4 ^a^	8.3 ± 0.5 ^b^	7.4 ± 0.5 ^a^	0.0001
Length of *Tibia* bone, cm	10.48 ± 0.5 ^a^	11.0 ± 0.7 ^a^	11.1 ± 0.7 ^b^	0.01
Carcass bones without wings and legs, g	554.26 ± 40.2 ^a^	564.7 ± 53.2 ^a^	611.4 ± 49.6 ^b^	0.001
Breast muscle yield (%)	26.75 ± 2.57 ^a^	32.00 ± 0.79 ^b^	29.93 ± 1.60 ^a^	0.01
Leg muscle yield (%)	17.50 ± 1.63	19.21 ± 0.76	19.48 ± 0.35	ns
Abdominal fat yield (%)	1.16 ± 0.18 ^b^	0.92 ± 0.30 ^a^	1.00 ± 0.27 ^a^	0.01

^a,b^—means with dissimilar letters varied significantly. The significance was defined at *p* ≤ 0.05. ns—not significant. *n* = 3 (replicates of analysis). Means of 10 birds per treatment. Birds were randomly selected and euthanised at 42 days of age. CON—control group; MCFAs—broiler group fed with Medium Chain Fatty Acids; OAs—broilers group fed with Organic Acids.

**Table 7:** Quality parameters of broilers breast meat samples.

Groups	DM %	pH	Color Coordinates			DL %	WHC %	CL %	SF kg/cm	IF %	Ash %	Protein Content %
			L*	a*	b*							
CON	25.24 ± 0.46	5.96 ± 0.03	70.28 ± 1.89	13.18 ± 0.75	13.25 ± 1.86 ^a^	1.62 ± 0.18	64.73 ± 1.84 ^a^	12.77 ± 0.66	1.02 ± 0.15	1.56 ± 0.32	1.22 ± 0.05	22.47 ± 0.39
MCFs	25.11 ± 0.97	6.02 ± 0.11	69.44 ± 4.04	11.58 ± 0.77	15.20 ± 1.29 ^b^	2.05 ± 0.64	66.28 ± 2.03 ^b^	12.82 ± 3.71	0.90 ± 0.18	2.93 ± 0.64	1.46 ± 0.10	20.72 ± 0.58
OAs	25.41 ± 1.49	6.02 ± 0.12	68.39 ± 2.70	12.30 ± 1.13	12.00 ± 2.22 ^a^	1.83 ± 0.49	64.88 ± 1.79 ^a^	11.87 ± 2.55	1.55 ± 0.61	3.11 ± 0.93	1.33 ± 0.20	20.96 ± 1.01
*p*	ns	ns	ns	ns	0.01	ns	0.001	ns	ns	ns	ns	ns

Data values are expressed as means with the standard deviations (*n* = 10). ns—not significant. *n* = 3 (replicates of analysis). Birds were randomly selected and euthanised at 42 days of age. Mean values within each column with different letters are significantly different (*p* ≤ 0.05). DM—dry matter. L*—lightness, a*—redness, b*—yellowness. DL—Drip loss. WHC—Water holding capacity. CL—Cooking loss. SF—Shear force. IF—Intramuscular fat. CON—control group; MCFAs—broiler group fed with Medium Chain Fatty Acids; OAs—broiler group fed with Organic Acids.

## Data Availability

Not applicable.
